# Supplementary material for: Are carbon emissions trading and green financial instruments synergistic? -Comprehensive quantitative research based on content analysis
Source: PLoS One. 2024 Mar 7;19(3):e0298601. doi: 10.1371/journal.pone.0298601 (PMC10919601; doi:10.1371/journal.pone.0298601)
Supplement: S1 Data — (ZIP) [file pone.0298601.s001.zip › Data/地级市Z_F工作报告.zip!/░▓╗╒/╗╞╔╜╩╨/└┤╫╘╣1⁄2╓┌║┼í╛┬φ┐╦╩2╛▌═°í┐_2015.docx]

各位代表：

现在，我代表市人民政府，向大会作工作报告，请予审议，并请市政协委员和其他列席人员提出意见。

一、2014年工作回顾

刚刚过去的2014年，面对错综复杂的外部环境和经济下行压力，全市人民在省委、省政府和市委的坚强领导下，深入学习贯彻党的十八大、十八届三中、四中全会和习近平总书记系列重要讲话精神，紧紧围绕建设现代国际旅游城市目标，以“十大战略支撑点”为抓手，坚持稳中求进、改革创新，着力提质增效、转型升级，较好完成了市六届人大三次会议确定的工作任务。

一是经济保持平稳增长。认真落实省、市促进经济持续健康较快发展和大力发展民营经济各项政策措施，加大对实体经济支持力度、加快经济结构调整，有针对性地解决突出矛盾和问题，实现了经济社会持续稳步发展。初步核算，全市实现生产总值511亿元、同比增长8.2%，财政收入90.2亿元、增长11.4%，全社会固定资产投资551.7亿元、增长5.1%，社会消费品零售总额220亿元、增长12.6%，外贸进出口总额9.1亿美元、增长12.9%，市内金融机构贷款余额526.2亿元、增长11.5%，城镇常住居民人均可支配收入23600元、增长8.5%，农村常住居民人均可支配收入10900元、增长11.5%。

二是转型发展步伐加快。扎实开展“提质增效、转型升级推进年”活动。大力发展现代农业，全年实现农业增加值54.2亿元、同比增长3.5%，规上农产品加工企业产值190.5亿元、增长9.3%。新增土地流转面积8万亩、特色产业基地7.2万亩、农民专业合作社196家；茶叶产量3.3万吨、增长5.8%，清洁化加工生产线达80条，祁门红茶在渤海商品交易所挂牌上市。制定工业转型升级规划，大力发展新型工业、优势产业，全年净增规上工业企业22家，规上工业增加值130.7亿元、增长11%。新增院士工作站1家、博士后工作站1家、中国驰名商标1家、国家高新技术企业11家、省级工程技术研究中心5家、省级高新技术产业基地1个，全市战略性新兴产业产值140亿元、增长14%。科宏股份等3家企业在“新三板”挂牌。加大园区建设和产业招商力度，落实土地使用税差别化征收政策，清理处置闲置低效用地2805亩，全市园区完成固定资产投资165亿元，实现工业总产值506亿元，园区税收占全市工业税收70%以上。深入实施国家服务业综合改革试点，完成黄山现代服务业产业园规划编制，服务业增加值220亿元、增长8.2%。借势《大黄山》热播，加大旅游营销力度，成功举办2014中国黄山国际旅游节等活动，全年接待游客4165.1万人次、旅游总收入354.4亿元，分别增长11.6%和12.7%。古徽州文化旅游区晋级国家5A级景区，黄山风景区入选全球百佳可持续目的地，我市被评为2014年度最佳国际旅游度假目的地，并荣获中国摄影之乡称号。

三是改革开放成效显著。加快推进政府机构改革，全面完成海事、工商质监食品药品监管体制改革以及中心城区城管体制改革，基本完成全市事业单位分类工作。“两个中心、两个工作站”建设经验做法在全省推广。稳步实施工商登记制度改革，新登记各类企业增长25%。扎实开展农村土地承包经营权确权登记工作，黟县土地确权试点工作全面完成。在全省率先完成农村合作金融机构改制任务，积极探索农民资金互助社建设。制定出台《黄山市古民居原地保护利用产权转让管理暂行办法》等9个规范性文件，古民居流转改革有序推进。深入推进林权流转改革，江南林权交易所与东方园林实现股权合作，与省内13个市签署了会员制合作协议，覆盖全省的林权交易平台基本建成。不断扩大对外开放，成功举办“联合国教科文组织名录遗产与可持续发展黄山对话会”，台湾居民口岸落地签注获批启用，黄山屯溪机场升格为国际机场，恢复黄山至香港、新开黄山至高雄包机航线，黄山口岸出入境达9.8万人次。持续推进招大引强，卓达集团新材料科技产业园等重点招商项目开工建设，甲骨文和易安信教育培训中心、科大讯飞“智慧旅游”等项目加快推进，中建材集团新材料产业园暨研发中心、安徽新华发行集团皖新物流园、惠普（黄山）软件创新基地、新徽商创新园、青岛伟业“欧洲之星”欢乐谷、国家地理四季黄山3D影院等项目成功签约，与北京国际信托、中国电力投资集团、省属五大文化企业集团签订战略合作协议，全年新签项目到位资金120.2亿元，外商直接投资2.78亿美元。

四是城乡建设扎实推进。强力推进重点项目建设，京福高铁黄山段线路建设全线建成，黄杭铁路开工建设，黄山北站、歙县北站主体完工，客运枢纽启动建设。 龙门岭隧道及接线工程竣工，歙黟一级公路、第二污水处理厂等项目加快推进。市图书馆、徽州糕饼博物馆竣工运营，农产品批发市场一期基本建成。棚户区和老旧小区改造稳步推进，屯溪老街公房住户搬迁224户。中心城区完成投资200亿元、增长9.9%。区县城和建制镇建设步伐加快，屯光等8个镇跻身全国重点镇。全市城镇化率提高1.2个百分点，达到50%。扎实开展美好乡村建设和“三线三边”环境整治，36个重点示范村通过省级考核验收，我市包揽皖南片区考核前五名，徽州区、黄山区、屯溪区荣获“全省美好乡村建设先进区县”称号，西递、宏村、猴坑、山岔入选“中国名村”300强，美好乡村建设走在全省前列。

五是生态优势日益彰显。获批国家主体功能区建设试点示范、首批国家生态文明先行示范区建设地区。新安江流域生态补偿机制试点通过中期绩效评估，试点延续达成初步共识。新安江上游段综合治理基本完成，农村保洁和河面打捞常态化开展，52个生态补偿机制试点项目完成投资25亿元。组织实施太平湖国家重点支持生态环境保护项目，完成投资7.5亿元。深入实施千万亩森林增长工程和绿色质量提升行动，完成471个提升点建设，新建苗木基地3845亩，工程造林6.1万亩，黄山区、祁门县跻身首批省级森林城市。认真落实节能减排工作责任制，全面完成省政府下达的节能减排和大气污染防治目标任务。全市生态环境保护工作在全省继续保持领先，空气质量稳居全省第一。在省社科院2014年安徽省城市科学发展评价中，我市位列榜首，在中国社科院2014年中国生态城市竞争力排名中名列第二。

六是民计民生持续改善。全市财政民生支出115.1亿元，增长10.5%。精心组织实施36项省市民生工程，完成投资37.3亿元，荣获全省民生工程绩效考评先进市称号。“一卡通”发放惠农补贴资金6.8亿元，人均受益580元。城镇新增就业3.2万人，失业人员再就业1.5万人，城镇登记失业率3.7%。持续扩大社会保障覆盖面，五项社会保险参保93.5万人次，城乡居民社会养老保险参保81.2万人。提高企业退休人员养老金、城乡低保、重点优抚和五保对象补助标准，省内异地就医实现即时结算。新农合参合率101%，大病保险全面启动，城乡医疗救助10.5万人次，发放救助资金2611万元。开工建设公共租赁住房和棚户区改造住房7579套，基本建成6638套，中心城区符合条件的低保低收入住房困难家庭，全部入住保障性住房。扎实推进地质灾害隐患点治理，歙县金竹村、休宁县阳台村等重点地质灾害点避让搬迁基本完成。实施扶贫项目553个，完成投资5.1亿元，2.2万人实现稳定脱贫。

七是社会事业全面发展。全面启动徽州古建筑保护利用工作，提升整治古村落40个、修缮古建筑151幢，打造新业态150处，完成投资9.8亿元，新增中国历史文化名镇名村5个和中国传统村落25个，108个古村落入选全省首批传统村落，徽州府衙修复获中国建筑工程鲁班奖。新建、改扩建各类幼儿园17所，完成23所义务教育阶段学校标准化建设，新设立市新城实验学校，启动屯溪一中新校区建设。继续推进城乡义务教育经费保障机制改革，中心城区义务教育阶段公办学校实现“零收费”。市医院综合大楼加快建设，市二院主体工程基本完工，市中医院搬迁并投入使用，“新安名医堂”对外接诊。全面落实单独两孩政策，人口自然增长率控制在5.5‰以内。全民健身运动蓬勃开展，我市运动健儿在十三届省运会上取得较好成绩，吕秀芝在亚运会上勇夺金牌。全面落实安全生产责任制，16项指标控制在省下达范围之内。圆满完成第九届村委会换届选举工作，8个精品社区、70个试点村社区完成建设。深入推进领导干部接访下访，加大矛盾纠纷排查化解力度，做好信访积案化解工作，信访形势总体平稳。扎实开展“六五”普法，深入开展平安创建，荣获“全省社会治安防控体系建设先进市”和“全省社会治安综合治理先进市”称号。国防教育、国防动员、民兵预备役、人民防空和双拥优抚安置工作不断加强。妇女、未成年人、老年人、残疾人权益得到保障。哲学社会科学、文学艺术、新闻出版、邮政、统计、档案、地方志工作得到加强。气象、地震、防灾减灾工作取得新进展，民族宗教、外事侨务、对台等工作取得新成绩。

八是自身建设不断加强。扎实开展党的群众路线教育实践活动，认真践行“三严三实”，严格执行中央八项规定精神和省、市委有关规定，着力解决“四风”方面存在的突出问题。大力推进依法行政，坚持民主科学决策，出台了进一步规范政府系统重大事项决策行为的实施意见。全力推进市级行政权力清单、责任清单和涉企收费清单制度建设，完成国务院、省政府取消、下放行政审批事项衔接落实工作，全年受理各类行政审批及服务事项15.6万件，提前办结率达99.3％。自觉接受市人大及其常委会的法律监督、工作监督和市政协的民主监督，451件人大代表建议和政协提案全部办结。大力改进文风会风，大幅度精简会议、文件和事务性活动，坚持勤俭办一切事业，全市“三公”经费支出下降27.3%。完善公共资源交易监管服务平台建设，切实加强政府投资项目、政府性资金、政府性债务和土地出让等管理。严格落实党风廉政建设责任制，扎实开展廉政风险防控，强化行政监察和审计监督，严肃查处了一批违纪违法案件。

各位代表，过去的一年，我们能够战胜困难和挑战，取得来之不易的成绩，是省委、省政府和市委坚强领导的结果，是市人大及其常委会和市政协监督支持的结果，是全市人民凝心聚力、真抓实干的结果。在此，我代表市人民政府，向全市广大工人、农民、知识分子、干部，向驻黄解放军指战员、武警官兵和政法干警，向各民主党派、各人民团体和各界人士，致以崇高的敬意！向关心、支持黄山现代化建设的中央、省驻黄单位和海内外友好人士，向在我市创业的投资者、建设者，表示衷心的感谢！

在肯定成绩的同时，必须清醒认识到经济社会发展中存在的困难和问题。主要是：部分指标未实现预期目标，特别是固定资产投资，受房地产市场和政府投资影响，增速降幅比较大；实体经济不强、产业支撑不足、财政收入质量不高，经济持续增长的基础不够稳固，转型升级任务艰巨；城乡居民收入增长后劲不足，部分城市低收入群体和深山区、库区、老区群众生活依然困难，就业、教育、医疗等方面还存在一些薄弱环节；生态保护、防灾抗灾、安全生产和社会治理的任务仍然十分繁重；少数政府部门和工作人员工作谋划不深，推动落实不力，慵懒散拖等不作为现象还不同程度存在；各级政府运用法治思维和法治方式解决问题的能力和水平还需进一步提高。对此，我们一定高度重视，采取切实措施，认真加以解决，不辜负各位代表和全市人民的期望和重托。

二、2015年主要工作

今年是全面深化改革的关键之年，是全面推进依法治市的开局之年，也是全面完成“十二五”规划的收官之年。当前宏观形势依然严峻，世界经济仍处于金融危机后的调整和修复期，国内经济下行压力依然较大。在积极应对困难和挑战的同时，我们更要看到黄山发展面临的有利条件，随着京福高铁建成通车“高铁时代”的到来，随着“六大战略平台”政策红利的释放，随着国家促进旅游业改革发展政策的落实，特别是近些年“十大战略支撑点”强力推进，转型升级、加快发展的基础更加坚实。我们要善审大势、把握趋势，顺势而为、乘势而上，全面加快现代国际旅游城市建设步伐。

今年政府工作的总体要求是：全面贯彻落实党的十八大、十八届三中、四中全会、中央和省经济工作会议精神，按照市委五届八次全会的总体部署，坚持稳中求进工作总基调，坚持以提高经济发展质量和效益为中心，主动适应经济发展新常态，把培育壮大市场主体、推进产业转型升级放到更加重要位置，狠抓改革攻坚，突出创业创新，统筹城乡发展，改善民计民生，加强和创新社会治理，促进经济平稳健康发展和社会和谐稳定。

今年经济社会发展的主要预期目标是：全市生产总值增长8%，财政收入增长7%，全社会固定资产投资增长8%，社会消费品零售总额增长10%，城镇常住居民人均可支配收入增长10%，农村常住居民人均可支配收入增长11%，居民消费价格涨幅控制在3%以内，万元生产总值能耗、主要污染物排放总量控制在省政府下达目标以内。在实际工作中，力求发展得更快更好一些。

按照上述目标和要求，今年的政府工作必须重点把握好四个方面工作导向。

一是更加注重培育市场主体。进一步降低准入门槛、搭建服务平台，让更多有创业意愿的人都能实现创业的梦想；进一步加大招商引资力度，吸引更多外部优质资源参与到黄山的建设发展中，让外来投资者都能取得合理的回报。通过营造更加优越的政策环境和制度环境，在黄山大地上掀起大众创业、企业创新的热潮。

二是更加注重培育新增长点。围绕推进新型工业化、信息化、城镇化、农业现代化，积极适应新的消费趋势，立足资源、产业和区位优势，坚持调整存量和做优增量并举，加快推进现有产业提质增效、转型升级，大力发展与互联网经济、文化创意、健康养生、运动休闲等相关联的产业，加快打造支撑黄山未来发展新的经济增长点。

三是更加注重统筹城乡建设。按照建设现代国际旅游城市的要求，高起点、高标准抓好城乡规划、建设和管理，进一步完善城市功能、提升城市能级，塑造乡村特色、彰显徽风徽韵，让我们的城市更有品位，让我们的乡村更有特色，让这里望得见山、看得见水、记得住乡愁，努力打造世界级旅游度假目的地。

四是更加注重改善民计民生。在新常态下更好地统筹经济建设和社会建设，更好地统筹经济增长和民生改善，强化产业发展增加公共财力，鼓励创业创新带动增收致富，不断提升城乡居民收入水平，让大家在衣食住行中真切感受到发展带来的实惠，不断增强全市人民幸福感和自豪感。

重点做好以下九个方面工作：

（一）全力扩大有效投入，持续推动招大引强

坚持经济工作项目化、项目工作责任化，全力抓好重点项目建设，全面提升项目工作水平，切实发挥项目的支撑作用和投资的拉动作用。

加快推进重点项目建设。集中力量抓好600个以上年投资计划超千万元的重点项目建设，全年完成投资超500亿元。全力抓好重大基础设施项目，加快黄杭铁路、歙黟一级公路、宁国至黄山天然气支线工程、高铁片区基础设施等项目建设，继续做好济祁、芜黄高速公路黄山段前期工作，力争月潭水库、黄千高速开工建设，确保京福高铁、黄山高铁客运枢纽（一期）等项目建成运营。全力抓好重大产业类项目，开工建设中关村（黄山）科技园、颐高电子商务产业园、皖新物流园、中建材新材料产业园暨研发中心、中国物联网黄山研发基地等项目，确保黄山农产品物流园、昌辉新区、康师傅瓶装水、卓达新材料等项目建成运营。通过一批重大基础设施和产业类项目的实施，助推即期经济增长，并为黄山长远发展增添动力。

加大招商引资力度。坚持把招商引资作为壮大经济总量、增强发展后劲的主抓手，坚持把一流的资源配置给一流的企业，立足日益凸显的生态优势、资源优势和区位优势，围绕加快建设区域中心城市，围绕开发园区转型升级，大力开展优势资源招商、产业链招商，力争海亮集团农业开发、红星美凯龙、台湾宝岛城、华侨城东黄山旅游开发、中国人寿花溪饭店改造及养生项目、万达文旅综合体、黄山“阅生活”城市文化综合体、龙腾影视文化创意园等一批重点在谈项目早日签约落地，力争再引进一批新型工业、现代服务业、现代农业项目，力争全年新签亿元以上项目100个以上，其中工业项目20个以上，新签项目开工率50%以上，到位内资增长8%，外商直接投资增长5%。积极探索PPP模式，加快推进水、电、气、路等一批基础设施和公共服务设施合作类项目建设。进一步完善重大招商项目联合预审机制、调度督查机制、考核奖惩机制，营造全市上下齐心合力抓招商的浓厚氛围。

切实提高项目工作水平。围绕皖南国际文化旅游示范区、国家主体功能区建设试点示范、国家生态文明先行示范区等战略平台，进一步加大政策研究力度，精心组织、科学编制“十三五”规划，谋划一批有质量、有深度、牵动力强的产业类和重大基础设施类项目，力争有更多的项目列入国家和省重大规划。按照依法合规、程序完善的要求，严格落实政府投资项目、工程招投标、土地出让等管理规定，加强对项目建设、资金使用、工程质量的全过程监管，切实提高政府投资效益。进一步完善市级领导联系重点项目制度，健全推进机制，全面提升项目服务水平。

（二）突出抓好产业发展，加快推进转型升级

坚持做精一产、做强二产、做优三产，切实把加快产业结构调整、培育新的增长点作为推动经济持续健康发展的主攻方向，增强产业核心竞争力。

大力发展现代农业。坚定不移加快转变农业发展方式，着力提升农业特色化、品牌化、规模化经营水平，力争农业增加值增长3.5%，规上农产品加工产值突破200亿元，增幅超过10%。打好生态牌、绿色牌，继续把茶产业作为发展现代农业的突破口，全年新发展无性系良种茶园1.5万亩、高效生态茶园3万亩，建成国家茶叶及农产品检测重点实验室，支持龙头企业加强基地建设、质量安全、品牌塑造、并购扩张、市场营销，举办祁门红茶、太平猴魁“百年金奖”系列活动，扩大黄山茶的品牌影响力；大力发展林下经济，推动毛竹、油茶、枇杷、菊花、山核桃、食用菌以及泉水鱼、皖南花猪等特色种养殖规模化经营，加强地理标志证明商标培育，力争全年新发展各类特色基地4.7万亩。大力开展农超对接，积极发展农产品电子商务，力争全年电子商务交易额突破5亿元；积极推广减肥降药等新技术，普及良种良法、测土配方、标准化养殖等实用技术，大力发展生态循环农业。深入推进土地流转，积极培育农业新型经营主体，新发展各类家庭农场80个以上，新增市级以上示范合作社30家。加强农田水利建设，提高农业防灾减灾能力。强化重大动植物疫病防控，确保主要鲜活农产品质量安全监测合格率稳定在98%以上。

加快发展工业经济。以《黄山市工业转型升级规划》为引领，优先发展绿色食品、电子信息、文化创意三大成长产业，加快打造装备制造、绿色软包装两大优势产业，改造提升精细化工、纺织服装两大传统产业，大力培育和发展生物医药、新材料等战略性新兴产业。加强对重点行业、规上企业的运行监测和协调服务，支持重点骨干企业兼并重组、加快扩张，支持中小企业协作配套、加快发展，全年净增规上工业企业30家，规上工业增加值增长11%。着力抓好127个重点技改项目，力争全年完成工业投资100亿元以上。充分发挥国家级企业技术中心、科技孵化器等创新平台作用，支持企业开展产学研合作，全年新认定国家高新技术企业10家，组建省级院士工作站1家、工程技术研究中心4家。坚持产城一体化发展方向，深入实施“百亿园区培育行动计划”，继续抓好园区“双清双控”，鼓励园区特色化、差异化发展，全市园区工业总产值增长10%以上、税收增长12%以上。加快机电、化工、纺织服装和农产品出口基地建设，鼓励企业积极开拓非洲、东盟、南美等新兴市场，全年外贸进出口总额增长8%。

持续推进旅游业转型升级。认真落实国务院、省政府关于促进旅游业改革发展实施意见，大力推进旅游、文化、生态三位一体发展，加快皖南国际文化旅游示范区建设，力争全年游客接待量增长12%，旅游总收入增长13%，入境游客增长10%，旅游创汇增长11%。突出抓好黄山风景区国际精品景区建设，巩固提升古徽州文化旅游区5A创建成果，深入推进齐云山生态文化旅游示范区建设，启动太平湖、齐云山5A创建工作。大力培育休闲度假、康体养生等体验性、参与性新型业态，推进旅游与文化、体育深度结合，采取市场化的办法，打造一批在全国有影响力的摄影、户外运动等品牌赛事，开发一系列既体现徽州文化、又有时尚特征的旅游商品。结合美好乡村建设和徽州传统村落保护，推出一批与国际接轨的高端乡村旅游度假产品。强化旅游目的地整体营销理念，完善旅游营销政策，创新营销方式，抢抓京福高铁开通和黄山口岸扩大对外开放机遇，重点拓展京福高铁沿线和东南亚等客源市场。启动外国人口岸落地签证前期工作，争取开通马来西亚、泰国包机航线。加快推进旅游信息化建设，组建黄山“国家自然景区智能旅游服务工程技术研究中心”，鼓励旅游企业大力发展电子商务，确保讯飞爱途智慧旅游上线运营。继续开展旅游市场专项整治，努力营造安全、优质、舒适的旅游环境。

着力提升服务业发展水平。全面启动黄山现代服务业产业园建设，加快园区基础设施建设，抓好园区产业规划和产业招商，努力打造新的经济增长点，力争全市服务业增加值增长8%。充分发挥服务业发展引导资金杠杆作用，积极发展现代物流、信息软件、服务外包、研发设计、商务会展等新兴服务业，鼓励支持重点服务业企业做大做强。加快推进“欧洲之星”欢乐谷、苏宁生活广场、河马动漫、奇瑞黄山汽车露营地等项目建设，力争安邦国际养生度假区、奥运户外运动基地、国家地理四季黄山3D影院等项目开工建设。合理引导商品住房消费，促进房地产市场健康发展。制定出台电子商务发展实施意见，组织实施国家电子商务进农村综合示范试点，借助互联网这一新兴平台，让更多的黄山特色产品走向全国、走向世界。

（三）统筹推进城乡发展，抓好城镇和美好乡村建设

加快推进以人为核心的新型城镇化，积极争创国家生态园林城市、“联合国人居奖”城市。

全力抓好城镇建设。启动“十三五”城市近期建设规划（2016－2020）编制，推进市县“多规合一”。重点抓好“一环三片”规划建设，加快建设新区快捷路、梅林南路，构筑城市外环，推进北部经济开发区和高铁站前区、西部现代服务业园区、东部醉温泉至花山谜窟高端休闲旅游区建设，进一步拉开城市框架。启动中心城区关键节点道路交口优化提升工作，完善城区路网，加快雨污分流改造，推进市生活垃圾处理场搬迁。按照“成熟一片、改造一片”的原则，继续推进仙人洞南路、荷花池等老旧小区和棚户区改造项目，启动屯溪老街新一轮改造提升。以正在实施的项目为牵引，每个区县选择2～3个建制镇，整合资源、完善配套，努力打造一批新型特色城镇。力争全市城镇化率提高1个百分点。扎实开展国家智慧城市试点，建成全市统一的城市数据中心。强化交通秩序和市容市貌整治，规范小区物业和各类市场管理，提升城市规划建设管理水平。

全面推进美好乡村建设。巩固扩大美好乡村建设成果，建立健全长效管护机制，全面推进51个省级中心村、19个市级中心村建设，扎实抓好271个自然村治理改造，规范管理世行贷款新农村建设项目，深化与32家省属企业结对共建，确保美好乡村建设继续走在全省前列。继续开展“三线三边”环境整治，确保高铁沿线、乡镇政府所在地和省市交界处环境有明显提升。坚持设施建设和产业发展并重，实施精准扶贫，突出抓好40个市级贫困村和纳入省“千村整推”工程计划贫困村的扶贫攻坚，力争减贫人口15%以上。

（四）全面深化各项改革，不断增强发展动力

坚持问题导向，解放思想、积极探索，深入推进重要领域和关键环节改革。

抓好各项改革举措落实。按照中央和省、市委的统一部署，扎实推进政府机构改革，进一步精简机构、转变职能。继续做好国务院、省政府取消和下放行政审批事项的衔接、落实工作，实施涉企收费清单制度，建立权力清单、责任清单制度。深化行政审批权相对集中改革，实现全市90%以上行政审批主体向一个内设机构集中、机构向各级政务服务中心集中。推进综合执法，减少市县两级政府执法队伍种类。深化财税体制改革，全面落实省以下财政体制改革和营改增等税制改革任务。分步实施并完成公务用车改革。完善事业单位分类改革配套政策，有序推进市直事业单位分类改革。全面推进医药卫生体制改革，启动城市公立医院改革。深化户籍制度改革，扎实推进新型城镇化试点。积极推进农村土地确权登记发证试点工作。

积极探索特色改革。深入实施国家服务业综合改革试点，推动传统服务业向现代服务业转变。扎实做好新安江流域生态补偿机制试点延续工作，力争试点期限延长、补偿标准提高、补偿范围拓展。以制度创新为核心，扎实推进国家生态文明先行示范区建设。深化林权制度改革，拓展江南林权交易所服务功能，加快建设立足黄山、面向全省、辐射全国的统一、规范、公开的区域性森林资源交易平台。深化市属国有企业改革，完善法人治理结构，切实增强市场竞争力。分类推进市直行政事业单位经营性资产归集工作，加大国有资源整合力度，提高国有资产保值增值率。坚持政府主导、市场运作，积极探索徽州古民居市场化保护利用的新思路、新办法，建立健全保护利用的新机制、新模式。

（五）持续改善生态环境，永葆一方蓝天碧水

认真贯彻执行新《环保法》，以国家主体功能区建设试点示范、国家生态文明先行示范区建设为契机，加快建设美丽中国先行区。

深入实施流域综合治理。认真落实国家《千岛湖及新安江上游流域水资源与生态环境保护综合规划》，在继续推进20项重点治理工作的基础上，更加突出面源污染治理，全年实施综合治理项目116个、完成投资108亿元，其中试点项目投资20亿元。深入实施太平湖国家重点支持生态环境保护项目，确保全年完成投资5.6亿元。扎实开展中小河流治理重点县综合整治及水系连通工程试点，加强水土流失治理。加大地质灾害隐患点排查和治理力度，积极推进地质灾害防治高标准“十有县”建设。扎实开展绿色质量提升行动，组织实施千万亩森林增长工程，完成新造林4万亩。突出抓好松材线虫病防控，深入发动群防群控，确保黄山松安全。加强城镇绿化的规划与建设，继续创建省级森林城市、森林城镇和森林村庄，确保创建国家森林城市通过验收。

突出抓好资源节约集约利用。深入开展环保专项行动，切实维护群众环境权益。继续推进大气污染防治工作，在全省率先实现大气质量监测县级全覆盖，确保大气环境质量在全省保持领先地位。规范设置和管理采石场、洗沙场，依法取缔破坏生态、污染环境的非煤矿山。深入开展集中式饮用水水源地环境综合整治。严格执行“三同时”制度，加强项目建设的全过程环境监管。严格落实节能减排目标责任制，全面完成省政府下达的目标任务。严格保护国土资源，确保全市耕地保有量和基本农田保护面积不减少，力争土地矿产卫片执法检查“零约谈、零问责”。继续抓好闲置低效土地清理处置工作，严格土地出让和储备管理，提高土地利用科学化、精细化水平。

（六）推进文化传承创新，增强文化软实力

大力弘扬徽州文化，充分挖掘潜力、激发活力，推动文化繁荣发展。

加大徽文化保护利用力度。深入实施徽州古建筑保护利用工作，启动38个重点保护项目，在巩固提升古村落、古民居、古书院、古道等保护利用成果的基础上，推进古祠堂、古桥、古塔、名人故居等修复保护。建立产权流转交易平台，鼓励社会力量认租、认购古民居，开办客栈、酒吧、文化场所等，让传统民居活起来、留下来。推进徽州文化生态保护区建设，继续开展可移动文物普查，出版发行《徽州文化大辞典》，加强博物馆集群建设，力争国家非遗展示基地、黟县国家历史文化名城申报成功。

发展壮大文化产业。续建新建重点文化产业项目100个以上，完成投资90亿元，力争文化产业增加值增长18%。加快推进徽州文化与生态长廊、徽文化产业园等16个文化产业集聚区建设，支持市文投公司等重点文化企业做大做强，推进省属文化企业与徽州古村落结对共建，新增省级文化产业示范基地3～5个。推进徽州四雕、文房四宝、万安罗盘、徽州漆器等规模化生产，组织非遗项目、民俗活动走进景区景点。加快摄影、演艺、写生基地建设，推动文化创意、动漫影视、教育培训等新兴产业发展。

提升公共文化服务水平。深入实施文化惠民工程，促进公共文化服务标准化、均等化。支持歙县开展省级公共文化服务体系示范区创建工作，全市新增农民文化乐园建设试点10～15个，完成402个广播电视村村通工程建设任务。以争创国家一级馆为目标，提升市文化馆、徽州文化博物馆、市图书馆软硬件水平。启动建设青少年科技馆、儿童乐园。举办市首届艺术节，推出一批精品力作。加强文化市场管理，为人民群众提供健康丰富的精神文化生活。

（七）抓好财政金融工作，增强发展保障能力

进一步优化财政收支结构，多渠道拓展金融资源，全面提升财政金融服务水平。

着力提高财政运行质量。用足用好营改增过渡期政策，整合并完善市级产业财政扶持政策，引入竞争性分配机制提升财政资金使用效果，多措并举促进财源建设。多方挖掘潜力，强化征收管理，把好收入质量关，提高税收占财政收入比重。盘活存量资金，完善政府预算体系，集中力量办大事。牢固树立长期过“紧日子”思想，硬化预算约束，继续压缩“三公”经费，一般公用经费实行“零增长”。建立规范的政府举债融资机制，将政府债务分类纳入全口径预算管理，建立健全债务风险预警及考核问责机制。

着力强化金融服务工作。把服务实体经济特别是中小企业作为信贷工作的重中之重，充分利用各项政策，努力做大总量、盘活存量、用好增量，力争市内金融机构贷款余额突破580亿元，全年贷款增幅达到全国、全省平均水平，社会融资总量增长12%以上。积极开展直接融资创新，通过企业债、中期票据、非公开定向发行等多种模式，增强企业和项目融资的多样性、灵活性。做好黄山旅游股份定向增发计划报批和发行，力争2～3户企业在“新三板”挂牌上市，市城投公司二期企业债券获批发行。大力发展普惠金融，推进农村金融综合服务站建设，推动市及区县供销农副产品专业合作社规范开展信用合作，支持银行机构开展面向农村的信贷业务，着力解决农户贷款难问题。加快发展地方金融组织，实现村镇银行县域全覆盖，积极引进股份制商业银行在我市设立分支机构。加强融资担保体系建设，规范发展小额贷款公司和融资性担保公司。推进社会信用体系建设，健全完善风险预警和应急处置协调机制，严厉打击各类非法集资行为，有效防范和化解金融风险。

（八）坚持以人为本，全力改善民计民生

按照“守住底线、突出重点、完善制度、引导舆论”的基本思路，加强保障和改善民生工作，让发展成果更多更公平惠及全市人民。

认真组织实施民生工程。坚持量力而行、尽力而为，在实施好省定民生工程建设任务的基础上，广泛征求各方面意见，梳理和安排好市级民生工程项目。加强项目资金和质量监管，加大管养资金整合力度，确保发挥持久效益，确保全市民生工程工作继续走在全省前列。

加强就业和社会保障工作。启动市人力资源市场建设，研究制定促进大众创业政策措施，深入开展就业帮扶和技能培训，统筹推进各类重点群体就业，确保全年城镇新增就业2.1万人，城镇登记失业率控制在4.2%以内。继续做好城乡养老保险制度衔接，落实社会保险关系转移接续政策，加大社会保险扩面征缴力度，加快金融功能社保卡发放工作，强化社保基金监管，确保全市五项社会保险参保超过90万人次，城乡居民社会养老保险参保达76万人。进一步完善城乡医疗救助制度，提高新农合筹资标准和受益水平，推进按病种付费和大病保险工作。做好城乡低保和农村五保工作，确保城乡低保标准和人均补差水平、农村五保供养标准年增长不低于10%。推进养老服务体系建设，开展居家养老服务，完善日间照料中心运营试点工作。推进住房公积金归集扩面，继续做好廉租房、公租房并轨运行和分配管理工作，完善住房保障制度，建成安置房1586套、公共租赁住房和棚户区改造住房7650套。

大力发展社会事业。扎实推进创新型城市建设。继续实施第二期学前教育三年行动计划，启动中小学教师无校籍管理改革，抓好屯溪一中等学校的联动搬迁，整合中心城区职业教育资源，集中建设一所具有一定规模的市属中等职业学校。推进黄山职业技术学院与企业开展联合办学。支持黄山学院建设有特色、高水平的应用型大学。加快推进市医院综合大楼、市疾控中心业务楼项目，提升“新安名医堂”诊疗水平，打响新安医学特色品牌。广泛开展全民健身运动，积极做好十四届省运会备战工作。深入开展爱国卫生运动，抓好重大疾病防控。继续稳定适度低生育水平，人口自然增长率控制在5.2‰以内。推动军民融合式发展，加强国防教育、国防动员、后备力量建设和人民防空工作，争创“全国双拥模范城”四连冠。充分发挥工会、共青团、妇联等人民团体的重要作用。切实维护妇女、儿童和老年人、残疾人合法权益。做好民族、宗教、统计、审计、物价、档案、地方志等工作。培育和践行社会主义核心价值观，努力争创全国文明城市。

（九）创新社会治理，维护社会和谐稳定

全面加强和创新社会治理方式，切实提高社会治理科学化水平，努力实现人民安居乐业、社会安定有序。

加强和创新社会治理。启动建设社会服务管理信息化平台，完善社区服务体系，方便群众办事。做好城市社区换届选举工作，打造一批新型农村社区。加强人民调解、行政调解、司法调解联动的衔接配合机制，做好社区矫正和安置帮教工作。深入开展“六五”普法，深化法治县（区）、民主法治示范村（社区）创建活动。完善法律援助制度，推进公共法律服务体系建设。健全社会稳定风险评估机制，提升“两个中心、两个工作站”建设水平，严格落实领导干部接访下访制度，积极推进网上受理信访事项，加大矛盾纠纷排查和信访积案化解力度。完善立体化社会治安防控体系，加强网络舆情监测，推进反恐应急机制建设，有效预防和严厉打击违法犯罪活动，确保社会大局和谐稳定。

加强公共安全体系建设。全面落实安全生产责任制，进一步强化政府监管职责，落实企业主体责任，突出抓好旅游、道路交通、非煤矿山、建筑施工、特种设备、烟花爆竹、危险化学品、地下油气管网、人群密集场所、古民居古建筑等重点领域专项整治，加强建设项目安全设施“三同时”监督管理，力保各项安全生产指标控制在省下达范围之内。切实做好防汛抗旱、森林防火和气象、地震等防灾减灾工作。加大食品药品安全监管力度。加强应急管理，强化应急专业队伍建设，推进重点行业领域视频监控联网，启动建设全市统一的应急指挥中心，提高应急保障能力。

三、切实加强政府自身建设

适应经济发展新常态，实现黄山发展新跨越，对政府工作提出了新的更高要求。我们必须顺势而为、主动作为、奋发有为。

一要强化发展意识，在新常态下有新业绩。发展是第一要务，是解决一切问题的基础和关键。面对新常态，我们必须坚持发展不动摇，始终牢记发展不足、发展不优是我市最大的市情，始终牢记实现更好更快发展是全市人民的共同愿望，以强烈的使命担当，昂扬的精神状态，务实的工作举措，在确保质量效益的前提下，争取发展得更快一些。面对新常态，必须胸怀全局善谋事，充分认识新常态带来的新变化，全面把握新变化中蕴含的新机遇，在全国全省发展的大格局中审视对标、谋划发展，切实提高工作的敏锐性、预见性和实效性，抢占发展制高点，打造发展新优势。面对新常态，必须扑下身子抓落实，坚决纠正懒政、怠政和为官不为的现象，坚决消除“只要不出事、宁愿不做事”的消极心态，坚决纠正“象征性执行”、“选择性执行”等不良作风，大力发扬“钉钉子”精神，对符合黄山实际、顺应群众期盼，看准了定下来的事，都要有专人去管去盯去促去干，真正把各项决策部署转化为实实在在的发展成效。

二要强化法治意识，在新形势下有新水平。党的十八届四中全会吹响了全面推进依法治国的新号角，我们必须按照中央和省、市委的决策部署，大力推进依法治市。加快建立权责统一、权威高效的依法行政体制，切实做到法定职责必须为、法无授权不可为。进一步规范重大事项决策行为，严格执行公众参与、专家论证、风险评估、合法性审查和集体讨论决定等法定程序，确保决策制度科学、程序正当、过程公开、责任明确。严格规范公正文明执法，完善行政执法程序，加强行政执法队伍建设，提高行政执法水平。推进行政复议委员会试点工作，依法办理各类行政复议案件。自觉接受人大及其常委会的法律监督、工作监督和政协的民主监督，认真听取人大代表、民主党派、工商联、无党派人士和各人民团体的意见。进一步加大政府信息公开和办事公开力度，主动接受社会公众和新闻舆论监督，让权力始终在阳光下运行。

三要强化公仆意识，在新期盼下有新作为。坚持把群众路线作为根本工作路线，进一步强化宗旨意识，始终秉持为民尽责的公仆情怀，巩固提升党的群众路线教育实践活动成果，健全深入了解民情、充分反映民意、广泛集中民智的长效机制。时刻把群众的安危冷暖放在心上，把更多的精力、更多的财力向农村、向基层、向城乡困难群众倾斜，多办顺民意、解民忧、惠民生的实事好事。继续开展效能、政风、行风“三位一体”考核评议，加强政务服务中心建设，办好“市民连线”和“政风行风热线”，坚决纠正损害群众利益的不正之风，为群众和企业提供更加优质、高效、便捷的服务。

四要强化廉洁意识，在新要求下有新形象。反腐倡廉必须常抓不懈，拒腐防变必须警钟长鸣。严格执行中央八项规定精神和省、市委有关要求，认真落实党风廉政建设责任制，全面推进惩治和预防腐败体系建设，健全完善廉政风险责任落实、动态监控、预警处置和检查评估机制，从源头上预防和惩治腐败。进一步加强制度建设，严格落实项目招投标、政府采购、国有土地出让等公共资源交易管理规定，把权力关进制度的笼子里，用制度管权、按制度办事。强化经济责任、民生工程和重大投资项目审计，严肃查处各类违纪违法案件，以反腐倡廉的实际成效取信于民、造福于民。

各位代表！

共筑更美更富的黄山梦，是时代赋予我们的神圣使命。让我们紧密团结在以习近平同志为总书记的党中央周围，在省委、省政府和市委的坚强领导下，紧紧依靠全市人民，凝心聚力，开拓创新，为加快建设现代国际旅游城市、打造美丽中国先行区而努力奋斗！
